# Supplementary material for: Studying the Antifungal Effects of Ageratina adenophora (Sprengel) R. King and H. Robinson (=Eupatorium adenophorum Sprengel) as a Bio-Fumigant Plant Alone and in Combination with Biochar Against Pythium aphanidermatum (Edson) Fitz
Source: Plants (Basel). 2024 Dec 16;13(24):3511. doi: 10.3390/plants13243511 (PMC11678674; doi:10.3390/plants13243511)
Supplement: Supplementary file 1 [file plants-13-03511-s001.zip › plants-3245295-supplementary.pdf]

Table S1. Main compounds extracted of dried roots-stems from *Eupatorium adenophorum* with SPME at four times

Row	Components	Time (days)				
		Control	7	14	21	30
1	Isopropyl butyrate	0.80	0.90	1.10	1.40	1.00
2	Phenol	1.00	1.30	1.60	2.40	1.80
3	D-Limonene	2.90	1.00	1.20	1.60	0.90
4	Ocimene	0.10	0.40	0.70	10.20	0.30
5	Cyclopentene	2.00	2.10	2.40	2.80	1.20
6	Octatriene	0.50	1.40	1.50	2.50	0.40
7	Norbornane	2.70	3.20	5.40	8.70	6.50
8	Naphthalene	0.40	0.60	2.00	6.00	4.00
9	Benzenemethanol	1.30	1.80	2.20	2.80	0.30
10	Hexanoic acid	2.80	3.10	4.10	5.50	5.00
11	Dehydrothymol	2.30	2.90	3.10	3.30	1.00
12	Bornyl acetate	68.90	0.40	0.60	0.80	0.20
13	Undecanone	14.00	15.60	22.20	25.90	20.30
14	Pentylcyclohexanone	6.60	7.80	12.30	15.50	9.80
15	Furanone	1.70	1.60	1.80	2.00	1.40
16	Bourbonene	1.40	0.40	0.60	2.70	0.90
17	Methyl-methylhept	13.40	14.60	16.00	18.30	10.70
18	Bergamotol	0.90	1.10	1.30	1.40	1.20
19	Adamantane	3.00	3.60	4.00	9.20	7.10
20	Muurolene	2.50	2.70	3.10	4.20	3.50
21	Butanoic acid	2.20	1.70	2.10	2.50	1.10
22	Spathulenol	12.30	17.50	21.60	26.50	18.10
23	Humulene	0.80	1.20	2.20	2.70	1.50
24	Heneicosane	0.40	0.50	1.00	1.20	0.30
25	Octocrylene	1.50	1.10	1.80	7.70	3.90
26	Octane	0.30	0.50	0.80	0.90	0.20

Table S2. Main compounds extracted of dried leaves from *Eupatorium adenophorum* with SPME at four times

Row	Component	Time (days)				
		Control	7	14	21	30
1	Butyric Acid	1.50	1.10	1.70	2.10	2.00
2	alpha. -Pinene	0.10	0.40	10.6	18.9	14.0
3	Dimethyl-1,6-octadiene	0.50	0.80	1.00	5.00	3.20
4	p-Cymene	17.50	5.60	1.70	7.10	3.30
5	Cyclopentasiloxane	3.00	4.40	5.10	6.00	4.30
6	Linalool oxide	0.50	0.70	1.30	1.70	1.00
7	p-Cresol	0.80	1.20	5.30	9.60	3.30
8	Nonanone	0.50	0.90	10.20	10.90	0.80
9	Benzene, 1,2,3,4-tetramethyl	1.30	1.10	1.40	2.00	0.90
10	Acetyl-1-methylcyclohexene	0.60	0.30	0.70	0.90	0.50
11	Ethanone	0.30	0.40	0.70	1.10	0.90
12	Cyclohexene-1-methanol	1.30	3.00	6.00	7.30	4.20
13	Benzofuran, 4,7-dimethyl	0.70	1.40	1.50	1.70	1.10
14	Dehydrothymol	1.00	1.30	1.80	2.30	0.90
15	Hydroxy-3-methylacetophenone	0.50	1.10	1.80	11.30	11.00
16	Dehydroelsholtzia ketone	0.80	0.60	1.40	1.80	1.10
17	hexahydronaphthalene	1.70	3.00	3.70	6.20	4.70
18	Bergamotene	93.70	8.00	13.30	19.20	5.60
19	Methyl-2-methylene	9.50	10.40	12.40	59.40	43.60
20	Benzene,	92.00	158.0	127.8	134.0	118.0
21	Caryophyllene oxide	9.80	6.70	16.70	20.60	20.00
22	Salvial	1.10	7.00	14.20	17.30	9.40
23	Humulene epoxide	0.80	1.70	2.10	2.70	2.20
24	Bisabolone	2.00	0.60	0.90	1.10	0.30
